# *cis*-Tetra­aqua­bis­{5-[4-(1*H*-imidazol-1-yl-κ*N* ^3^)phen­yl]tetra­zolido}manganese(II) dihydrate

**DOI:** 10.1107/S1600536812010446

**Published:** 2012-03-17

**Authors:** Shao-Wei Tong, Wen-Dong Song, Dong-Liang Miao, Shi-Jie Li, Jing-Bo An

**Affiliations:** a College of Food Science and Technology, Guangdong Ocean University, Zhanjiang 524088, People’s Republic of China; b College of Science, Guangdong Ocean University, Zhanjiang 524088, People’s Republic of China; c School of Enviroment Science and Engineering, Donghua University, Shanghai 200051, People’s Republic of China

## Abstract

In the title compound, [Mn(C_10_H_7_N_6_)_2_(H_2_O)_4_]·2H_2_O, the Mn^2+^ lies on a twofold rotation axis and is six-coordinated by two N atoms from the *cis*-related monodentate 5-[4-(imidazol-1-yl)phen­yl]tetra­zolide ligands and four O atoms from the coordinated water mol­ecules. The complex mol­ecules are connected *via* water O—H⋯O and O—H⋯N hydrogen bonds and weak π–π stacking inter­actions between the benzene rings [minimum ring centroid separation = 3.750 (6) Å] into a three-dimensional polymeric structure. The imidazolyl group of the ligand is partially disordered over two sets of sites with refined occupancies of 0.531 (7):0.469 (7).

## Related literature

For our previous work based on imidazole derivatives as ligands, see: Li, Song *et al.* (2011 ▶); Li, Ma *et al.* (2011 ▶); Fan *et al.* (2010 ▶); Li *et al.* (2010 ▶). For related structures, see: Huang *et al.* (2009 ▶); Cheng (2011 ▶). An independent determination of the title structure is reported by Wang *et al.* (2012 ▶).
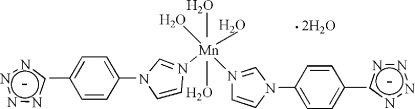


## Experimental

### 

#### Crystal data

[Mn(C_10_H_7_N_6_)_2_(H_2_O)_4_]·2H_2_O
*M* *_r_* = 585.47
Monoclinic, 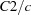

*a* = 19.1342 (18) Å
*b* = 13.2100 (4) Å
*c* = 13.3280 (13) Å
β = 131.056 (2)°
*V* = 2540.3 (4) Å^3^
*Z* = 4
Mo *K*α radiation
μ = 0.58 mm^−1^
*T* = 294 K
0.80 × 0.11 × 0.10 mm

#### Data collection

Rigaku/MSC Mercury CCD diffractometer
Absorption correction: multi-scan (*SADABS*; Sheldrick, 1996 ▶) *T*_min_ = 0.653, *T*_max_ = 0.944
8421 measured reflections
2239 independent reflections
1957 reflections with *I* > 2σ(*I*)
*R*_int_ = 0.042

#### Refinement

*R*[*F* ^2^ > 2σ(*F* ^2^)] = 0.056
*wR*(*F* ^2^) = 0.142
*S* = 1.31
2239 reflections
196 parameters
512 restraints
H-atom parameters constrained
Δρ_max_ = 0.34 e Å^−3^
Δρ_min_ = −0.55 e Å^−3^

### 

Data collection: *RAPID-AUTO* (Rigaku/MSC, 1998) ▶; cell refinement: *RAPID-AUTO*; data reduction: *CrystalStructure* (Rigaku/MSC, 2002 ▶); program(s) used to solve structure: *SHELXS97* (Sheldrick, 2008 ▶); program(s) used to refine structure: *SHELXL97* (Sheldrick, 2008 ▶); molecular graphics: *SHELXTL* (Sheldrick, 2008 ▶); software used to prepare material for publication: *SHELXTL*.

## Supplementary Material

Crystal structure: contains datablock(s) I, global. DOI: 10.1107/S1600536812010446/zs2182sup1.cif

Structure factors: contains datablock(s) I. DOI: 10.1107/S1600536812010446/zs2182Isup2.hkl

Additional supplementary materials:  crystallographic information; 3D view; checkCIF report

## Figures and Tables

**Table 1:** Hydrogen-bond geometry (Å, °)

*D*—H⋯*A*	*D*—H	H⋯*A*	*D*⋯*A*	*D*—H⋯*A*
O3—H3*E*⋯N5^i^	0.85	2.65	3.397 (4)	147
O3—H3*E*⋯N6^i^	0.85	1.89	2.726 (4)	169
O3—H3*D*⋯N4^ii^	0.85	2.63	3.261 (5)	132
O3—H3*D*⋯N3^ii^	0.85	1.95	2.774 (5)	162
O2—H2*D*⋯O3^iii^	0.85	1.84	2.684 (4)	170
O2—H2*C*⋯O3	0.85	1.90	2.745 (4)	170
O1—H1*D*⋯N5^iv^	0.85	1.96	2.811 (4)	179
O1—H1*C*⋯N5^v^	0.85	2.62	3.396 (4)	152
O1—H1*C*⋯N4^v^	0.85	1.99	2.835 (4)	179

Symmetry codes: (i) 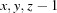; (ii) 
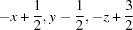
; (iii) 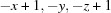; (iv) 
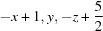
; (v) 
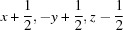
.

**Fig. 1.**
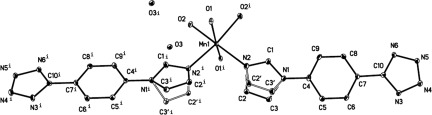
The molecular configuration and atom numbering scheme of the title compound, with non-H atoms shown as 30% probability displacement ellipsoids. For symmetry code (i): -x + 1, y, -z + 3/2.

## supplementary crystallographic information

### Comment

In recent years, our research group has shown great interest in the design and synthesis of interesting metal–organic comlexes with imidazole derivatives such as 2-propyl-imidazole-4,5-dicarboxylic acid (Fan *et al.*, 2010; Li *et al.*, 2010) and 2-ethyl-1*H*-imidazole-4,5-dicarboxylic acid (Li, Song *et al.*, 2011; Li, Ma *et al.*, 2011). In this paper, we report the synthesis and structure of a new Mn^II^ complex, [Mn(C_10_H_7_N_6_)_2_(H_2_O)_4_] . 2(H_2_O) and the structure is reported here.

As shown in the Fig. 1, the title complex molecule comprises the Mn^2+^ ion which lies on a crystallographic twofold rotation axis and is six-coordinated by two N atoms from the *cis*-related monodentate 5-[4-(imidazol-1-yl)phenyl]tetrazolide ligands and four O atoms from the coordinated water molecules. The complex has a slightly distorted octahedral geometry [Mn—N = 2.256 (4) Å; Mn—O = 2.177 (3) and 2.204 (3) Å]. In the crystal structure, the complex molecules are connected *via* water O—H···O and O—H···N hydrogen bonds (Table 1) into a three-dimensional supramolecular structure which is further stabilized by weak π–π stacking interactions between benzene rings [minimum ring centroid distance, 3.750 (6) Å]. The atoms C2 and C3 of the imidazolyl ring of the ligand are disordered over two sites (C2' and C3') with refined occupancies of 0.531 (7):0.469 (7), respectively. The structure of the anhydrous *trans* isomer of this complex has previosly been reported (Cheng, 2011).

### Experimental

A mixture of manganese(II) chloride (0.1 mmol, 0.020 g) and 5-[4-(imidazol-1-yl)phenyl]tetrazole (1-tetrazole-4-imidazole-benzene) (0.2 mmol, 0.043 g) in 15 ml of water was sealed in an autoclave equipped with a Teflon liner (25 ml) and then heated at 413 K for 3 days. Crystals of the title compound were obtained by slow evaporation of the solvent at room temperature.

### Refinement

H atoms of the water molecule were located in a difference-Fourier map and refined as riding with an O—H distance restraint of 0.85 Å, with *U*_iso_(H) = 1.5 *U*_eq_. The imidazolyl and phenyl H atoms were located in a difference-Fourier but were refined as riding with C—H = 0.93 Å and *U*_iso_(H) = 1.5*U*_eq_(C). The imidazolyl groups of the ligand are partially disordered over two sets of sites (C2, C2' and C3, C3') with refined occupancies of 0.531 (7) : 0.469 (7).

### Crystal data

**Table:**

[Mn(C_10_H_7_N_6_)_2_(H_2_O)_4_]·2H_2_O	*F*(000) = 1212
*M**_r_* = 585.47	*D*_x_ = 1.531 Mg m^−^^3^
Monoclinic, *C*2/*c*	Mo *K*α radiation, λ = 0.71073 Å
Hall symbol: -C 2yc	Cell parameters from 3180 reflections
*a* = 19.1342 (18) Å	θ = 3.1–30.0°
*b* = 13.2100 (4) Å	µ = 0.58 mm^−^^1^
*c* = 13.3280 (13) Å	*T* = 294 K
β = 131.056 (2)°	Block, colourless
*V* = 2540.3 (4) Å^3^	0.80 × 0.11 × 0.10 mm
*Z* = 4	

### Data collection

**Table:**

Rigaku/MSC Mercury CCD diffractometer	2239 independent reflections
Radiation source: fine-focus sealed tube	1957 reflections with *I* > 2σ(*I*)
Graphite monochromator	*R*_int_ = 0.042
ω scans	θ_max_ = 25.0°, θ_min_ = 3.1°
Absorption correction: multi-scan (*SADABS*; Sheldrick, 1996)	*h* = −22→22
*T*_min_ = 0.653, *T*_max_ = 0.944	*k* = −15→15
8421 measured reflections	*l* = −15→15

### Refinement

**Table:**

Refinement on *F*^2^	Primary atom site location: structure-invariant direct methods
Least-squares matrix: full	Secondary atom site location: difference Fourier map
*R*[*F*^2^ > 2σ(*F*^2^)] = 0.056	Hydrogen site location: inferred from neighbouring sites
*wR*(*F*^2^) = 0.142	H-atom parameters constrained
*S* = 1.31	*w* = 1/[σ^2^(*F*_o_^2^) + (0.0076*P*)^2^ + 23.3787*P*] where *P* = (*F*_o_^2^ + 2*F*_c_^2^)/3
2239 reflections	(Δ/σ)_max_ < 0.001
196 parameters	Δρ_max_ = 0.34 e Å^−^^3^
512 restraints	Δρ_min_ = −0.55 e Å^−^^3^

### Special details

**Table:**

Geometry. All e.s.d.'s (except the e.s.d. in the dihedral angle between two l.s. planes) are estimated using the full covariance matrix. The cell e.s.d.'s are taken into account individually in the estimation of e.s.d.'s in distances, angles and torsion angles; correlations between e.s.d.'s in cell parameters are only used when they are defined by crystal symmetry. An approximate (isotropic) treatment of cell e.s.d.'s is used for estimating e.s.d.'s involving l.s. planes.
Refinement. Refinement of *F*^2^ against ALL reflections. The weighted *R*-factor *wR* and goodness of fit *S* are based on *F*^2^, conventional *R*-factors *R* are based on *F*, with *F* set to zero for negative *F*^2^. The threshold expression of *F*^2^ > σ(*F*^2^) is used only for calculating *R*-factors(gt) *etc*. and is not relevant to the choice of reflections for refinement. *R*-factors based on *F*^2^ are statistically about twice as large as those based on *F*, and *R*- factors based on ALL data will be even larger.

### Fractional atomic coordinates and isotropic or equivalent isotropic displacement parameters (Å^2^)

**Table:**

	*x*	*y*	*z*	*U*_iso_*/*U*_eq_	Occ. (<1)
Mn1	0.5000	0.14050 (7)	0.7500	0.0114 (2)	
N1	0.4195 (3)	0.3148 (3)	0.9401 (4)	0.0197 (8)	
N2	0.4707 (3)	0.2569 (3)	0.8420 (4)	0.0189 (8)	
N3	0.2780 (3)	0.3910 (3)	1.2697 (4)	0.0190 (8)	
N4	0.2668 (3)	0.3553 (3)	1.3534 (4)	0.0201 (8)	
N5	0.2945 (2)	0.2610 (3)	1.3831 (3)	0.0158 (8)	
N6	0.3247 (2)	0.2320 (3)	1.3206 (3)	0.0149 (7)	
O1	0.65066 (19)	0.1248 (2)	0.9144 (3)	0.0164 (7)	
H1C	0.6860	0.1315	0.8971	0.020*	
H1D	0.6680	0.1664	0.9757	0.020*	
O2	0.5016 (2)	0.0210 (2)	0.6390 (3)	0.0172 (7)	
H2C	0.4550	0.0260	0.5564	0.021*	
H2D	0.5483	0.0045	0.6477	0.021*	
O3	0.3656 (2)	0.0306 (2)	0.3671 (3)	0.0178 (7)	
H3D	0.3190	−0.0069	0.3354	0.021*	
H3E	0.3466	0.0912	0.3426	0.021*	
C1	0.4461 (3)	0.2365 (3)	0.9100 (5)	0.0227 (10)	
H1	0.4471	0.1708	0.9362	0.027*	
C2	0.4225 (6)	0.3504 (6)	0.7794 (8)	0.0186 (17)	0.531 (7)
H2	0.4149	0.3814	0.7102	0.022*	0.531 (7)
C3	0.3898 (6)	0.3863 (6)	0.8365 (8)	0.0184 (17)	0.531 (7)
H3	0.3553	0.4450	0.8139	0.022*	0.531 (7)
C2'	0.5005 (7)	0.3579 (7)	0.8818 (9)	0.0181 (19)	0.469 (7)
H2'	0.5348	0.3938	0.8669	0.022*	0.469 (7)
C3'	0.4721 (7)	0.3956 (7)	0.9450 (9)	0.0192 (19)	0.469 (7)
H3'	0.4840	0.4593	0.9828	0.023*	0.469 (7)
C4	0.3907 (3)	0.3145 (3)	1.0161 (4)	0.0148 (8)	
C5	0.3558 (3)	0.4030 (3)	1.0259 (4)	0.0172 (9)	
H5	0.3501	0.4616	0.9822	0.021*	
C6	0.3299 (3)	0.4027 (3)	1.1017 (4)	0.0178 (9)	
H6	0.3060	0.4613	1.1080	0.021*	
C7	0.3392 (3)	0.3158 (3)	1.1684 (4)	0.0133 (8)	
C8	0.3722 (3)	0.2276 (3)	1.1547 (4)	0.0153 (9)	
H8	0.3767	0.1684	1.1963	0.018*	
C9	0.3986 (3)	0.2275 (3)	1.0794 (4)	0.0180 (9)	
H9	0.4216	0.1687	1.0718	0.022*	
C10	0.3140 (3)	0.3136 (3)	1.2521 (4)	0.0139 (9)	

### Atomic displacement parameters (Å^2^)

**Table:**

	*U*^11^	*U*^22^	*U*^33^	*U*^12^	*U*^13^	*U*^23^
Mn1	0.0141 (5)	0.0116 (4)	0.0135 (5)	0.000	0.0113 (4)	0.000
N1	0.031 (2)	0.0127 (17)	0.031 (2)	0.0007 (15)	0.0271 (18)	−0.0012 (15)
N2	0.026 (2)	0.0149 (18)	0.0275 (19)	−0.0026 (16)	0.0226 (17)	−0.0031 (15)
N3	0.027 (2)	0.0169 (19)	0.026 (2)	0.0046 (16)	0.0230 (18)	0.0029 (15)
N4	0.029 (2)	0.0180 (18)	0.0255 (19)	0.0026 (17)	0.0233 (18)	0.0018 (16)
N5	0.0204 (19)	0.0150 (18)	0.0179 (18)	0.0006 (15)	0.0152 (16)	0.0009 (14)
N6	0.0191 (18)	0.0152 (18)	0.0150 (17)	0.0001 (15)	0.0132 (15)	0.0001 (14)
O1	0.0183 (15)	0.0209 (16)	0.0174 (15)	−0.0029 (13)	0.0148 (14)	−0.0036 (13)
O2	0.0157 (16)	0.0216 (16)	0.0178 (15)	0.0008 (13)	0.0124 (14)	−0.0021 (13)
O3	0.0195 (16)	0.0145 (15)	0.0229 (16)	0.0009 (13)	0.0155 (14)	−0.0001 (13)
C1	0.038 (3)	0.015 (2)	0.031 (2)	0.0024 (19)	0.030 (2)	−0.0001 (18)
C2	0.026 (4)	0.015 (4)	0.024 (4)	0.001 (3)	0.020 (3)	0.001 (3)
C3	0.025 (4)	0.012 (3)	0.026 (4)	0.002 (3)	0.020 (3)	0.001 (3)
C2'	0.028 (4)	0.013 (4)	0.024 (4)	−0.006 (3)	0.022 (3)	−0.003 (3)
C3'	0.026 (4)	0.018 (4)	0.024 (4)	−0.003 (3)	0.021 (3)	−0.001 (3)
C4	0.015 (2)	0.017 (2)	0.019 (2)	−0.0056 (16)	0.0138 (17)	−0.0053 (16)
C5	0.024 (2)	0.013 (2)	0.021 (2)	−0.0015 (17)	0.0177 (18)	0.0002 (17)
C6	0.022 (2)	0.016 (2)	0.024 (2)	0.0031 (17)	0.0188 (19)	−0.0001 (17)
C7	0.014 (2)	0.016 (2)	0.0128 (19)	0.0001 (16)	0.0102 (17)	−0.0004 (16)
C8	0.018 (2)	0.013 (2)	0.0155 (19)	−0.0002 (17)	0.0114 (17)	0.0010 (16)
C9	0.021 (2)	0.017 (2)	0.023 (2)	0.0031 (17)	0.0173 (18)	−0.0016 (17)
C10	0.014 (2)	0.0125 (19)	0.016 (2)	0.0001 (16)	0.0098 (17)	−0.0007 (16)

### Geometric parameters (Å, º)

**Table:**

Mn1—O2^i^	2.177 (3)	O2—H2D	0.8500
Mn1—O2	2.177 (3)	O3—H3D	0.8500
Mn1—O1	2.204 (3)	O3—H3E	0.8499
Mn1—O1^i^	2.204 (3)	C1—H1	0.9300
Mn1—N2	2.256 (4)	C2—C3	1.349 (11)
Mn1—N2^i^	2.256 (4)	C2—H2	0.9300
N1—C1	1.327 (6)	C3—H3	0.9300
N1—C4	1.436 (5)	C2'—C3'	1.361 (12)
N1—C3'	1.438 (10)	C2'—H2'	0.9300
N1—C3	1.446 (9)	C3'—H3'	0.9300
N2—C1	1.293 (5)	C4—C9	1.374 (6)
N2—C2'	1.410 (10)	C4—C5	1.393 (6)
N2—C2	1.436 (9)	C5—C6	1.389 (6)
N3—C10	1.336 (5)	C5—H5	0.9300
N3—N4	1.352 (5)	C6—C7	1.390 (6)
N4—N5	1.309 (5)	C6—H6	0.9300
N5—N6	1.346 (5)	C7—C8	1.393 (6)
N6—C10	1.338 (5)	C7—C10	1.478 (5)
O1—H1C	0.8500	C8—C9	1.388 (6)
O1—H1D	0.8501	C8—H8	0.9300
O2—H2C	0.8500	C9—H9	0.9300
			
O2^i^—Mn1—O2	87.07 (16)	H3D—O3—H3E	108.3
O2^i^—Mn1—O1	81.34 (11)	N2—C1—N1	115.9 (4)
O2—Mn1—O1	90.81 (11)	N2—C1—H1	122.0
O2^i^—Mn1—O1^i^	90.81 (11)	N1—C1—H1	122.0
O2—Mn1—O1^i^	81.34 (11)	C3—C2—N2	109.5 (7)
O1—Mn1—O1^i^	169.20 (16)	C3—C2—H2	125.3
O2^i^—Mn1—N2	90.29 (12)	N2—C2—H2	125.3
O2—Mn1—N2	169.50 (12)	C2—C3—N1	105.8 (7)
O1—Mn1—N2	98.84 (12)	C2—C3—H3	127.1
O1^i^—Mn1—N2	88.54 (12)	N1—C3—H3	127.1
O2^i^—Mn1—N2^i^	169.50 (12)	C3'—C2'—N2	110.6 (7)
O2—Mn1—N2^i^	90.29 (12)	C3'—C2'—H2'	124.7
O1—Mn1—N2^i^	88.54 (12)	N2—C2'—H2'	124.7
O1^i^—Mn1—N2^i^	98.84 (12)	C2'—C3'—N1	104.6 (7)
N2—Mn1—N2^i^	94.05 (18)	C2'—C3'—H3'	127.7
C1—N1—C4	127.8 (4)	N1—C3'—H3'	127.7
C1—N1—C3'	101.3 (5)	C9—C4—C5	120.7 (4)
C4—N1—C3'	123.5 (5)	C9—C4—N1	119.8 (4)
C1—N1—C3	102.0 (4)	C5—C4—N1	119.5 (4)
C4—N1—C3	125.7 (4)	C6—C5—C4	119.2 (4)
C3'—N1—C3	51.9 (5)	C6—C5—H5	120.4
C1—N2—C2'	100.2 (5)	C4—C5—H5	120.4
C1—N2—C2	101.3 (4)	C5—C6—C7	120.8 (4)
C2'—N2—C2	49.6 (5)	C5—C6—H6	119.6
C1—N2—Mn1	125.0 (3)	C7—C6—H6	119.6
C2'—N2—Mn1	131.7 (4)	C6—C7—C8	119.0 (4)
C2—N2—Mn1	124.4 (4)	C6—C7—C10	122.0 (4)
C10—N3—N4	104.9 (3)	C8—C7—C10	119.0 (4)
N5—N4—N3	109.2 (3)	C9—C8—C7	120.5 (4)
N4—N5—N6	109.8 (3)	C9—C8—H8	119.7
C10—N6—N5	104.8 (3)	C7—C8—H8	119.7
Mn1—O1—H1C	118.3	C4—C9—C8	119.8 (4)
Mn1—O1—H1D	108.9	C4—C9—H9	120.1
H1C—O1—H1D	108.4	C8—C9—H9	120.1
Mn1—O2—H2C	110.6	N3—C10—N6	111.3 (4)
Mn1—O2—H2D	125.2	N3—C10—C7	125.3 (4)
H2C—O2—H2D	108.1	N6—C10—C7	123.4 (4)
			
O2^i^—Mn1—N2—C1	−11.2 (4)	C1—N2—C2'—C3'	−13.7 (9)
O2—Mn1—N2—C1	64.1 (9)	C2—N2—C2'—C3'	82.6 (9)
O1—Mn1—N2—C1	−92.5 (4)	Mn1—N2—C2'—C3'	−173.9 (5)
O1^i^—Mn1—N2—C1	79.6 (4)	N2—C2'—C3'—N1	−1.9 (10)
N2^i^—Mn1—N2—C1	178.3 (5)	C1—N1—C3'—C2'	16.4 (8)
O2^i^—Mn1—N2—C2'	144.8 (6)	C4—N1—C3'—C2'	168.1 (6)
O2—Mn1—N2—C2'	−139.8 (8)	C3—N1—C3'—C2'	−80.2 (8)
O1—Mn1—N2—C2'	63.5 (6)	C1—N1—C4—C9	7.3 (7)
O1^i^—Mn1—N2—C2'	−124.4 (6)	C3'—N1—C4—C9	−136.7 (6)
N2^i^—Mn1—N2—C2'	−25.6 (6)	C3—N1—C4—C9	159.1 (5)
O2^i^—Mn1—N2—C2	−151.5 (5)	C1—N1—C4—C5	−173.4 (5)
O2—Mn1—N2—C2	−76.1 (9)	C3'—N1—C4—C5	42.6 (7)
O1—Mn1—N2—C2	127.2 (5)	C3—N1—C4—C5	−21.6 (7)
O1^i^—Mn1—N2—C2	−60.7 (5)	C9—C4—C5—C6	0.6 (7)
N2^i^—Mn1—N2—C2	38.1 (4)	N1—C4—C5—C6	−178.7 (4)
C10—N3—N4—N5	−0.3 (5)	C4—C5—C6—C7	0.6 (7)
N3—N4—N5—N6	0.1 (5)	C5—C6—C7—C8	−2.0 (7)
N4—N5—N6—C10	0.2 (4)	C5—C6—C7—C10	178.7 (4)
C2'—N2—C1—N1	27.0 (6)	C6—C7—C8—C9	2.3 (6)
C2—N2—C1—N1	−23.5 (6)	C10—C7—C8—C9	−178.5 (4)
Mn1—N2—C1—N1	−170.9 (3)	C5—C4—C9—C8	−0.4 (7)
C4—N1—C1—N2	−178.7 (4)	N1—C4—C9—C8	178.9 (4)
C3'—N1—C1—N2	−28.7 (6)	C7—C8—C9—C4	−1.0 (7)
C3—N1—C1—N2	24.4 (6)	N4—N3—C10—N6	0.5 (5)
C1—N2—C2—C3	12.3 (8)	N4—N3—C10—C7	−179.7 (4)
C2'—N2—C2—C3	−81.5 (8)	N5—N6—C10—N3	−0.4 (5)
Mn1—N2—C2—C3	160.0 (5)	N5—N6—C10—C7	179.7 (4)
N2—C2—C3—N1	1.1 (9)	C6—C7—C10—N3	2.2 (7)
C1—N1—C3—C2	−13.8 (7)	C8—C7—C10—N3	−177.1 (4)
C4—N1—C3—C2	−171.3 (5)	C6—C7—C10—N6	−178.0 (4)
C3'—N1—C3—C2	81.2 (8)	C8—C7—C10—N6	2.8 (6)

Symmetry code: (i) −*x*+1, *y*, −*z*+3/2.

### Hydrogen-bond geometry (Å, º)

**Table:**

*D*—H···*A*	*D*—H	H···*A*	*D*···*A*	*D*—H···*A*
O3—H3*E*···N5^ii^	0.85	2.65	3.397 (4)	147
O3—H3*E*···N6^ii^	0.85	1.89	2.726 (4)	169
O3—H3*D*···N4^iii^	0.85	2.63	3.261 (5)	132
O3—H3*D*···N3^iii^	0.85	1.95	2.774 (5)	162
O2—H2*D*···O3^iv^	0.85	1.84	2.684 (4)	170
O2—H2*C*···O3	0.85	1.90	2.745 (4)	170
O1—H1*D*···N5^v^	0.85	1.96	2.811 (4)	179
O1—H1*C*···N5^vi^	0.85	2.62	3.396 (4)	152
O1—H1*C*···N4^vi^	0.85	1.99	2.835 (4)	179

Symmetry codes: (ii) *x*, *y*, *z*−1; (iii) −*x*+1/2, *y*−1/2, −*z*+3/2; (iv) −*x*+1, −*y*, −*z*+1; (v) −*x*+1, *y*, −*z*+5/2; (vi) *x*+1/2, −*y*+1/2, *z*−1/2.
